# Prevalence and determinants of asphyxia neonatorum among live births at Debre Tabor General Hospital, North Central Ethiopia: a cross-sectional study

**DOI:** 10.4314/ahs.v21i1.49

**Published:** 2021-03

**Authors:** Wubet Alebachew Bayih, Tadesse Gashaw Tezera, Abebaw Yeshambel Alemu, Demeke Mesfin Belay, Habtamu Shimelis Hailemeskel, Metadel Yibeltal Ayalew

**Affiliations:** 1 College of Health Sciences, Debre Tabor University, P.O.BOX. 272, Debre Tabor, Ethiopia; 2 Tibebe Ghion Specialized Hospital, Bahir Dar University, Bahir Dar, Ethiopia

**Keywords:** Birth asphyxia, asphyxia neonatorum, prevalence, determinants, Ethiopia

## Abstract

**Background:**

More than one third of the neonatal deaths at Neonatal Intensive Care Unit of Debre Tabor General Hospital (DTGH) are attributable to birth asphyxia. Most of these neonates are referred from maternity ward of the hospital. However, there is no recent evidence on the prevalence and specific determinants of birth asphyxia at DTGH. Besides, public health importance of factors like birth spacing weren't addressed in the prior studies.

**Methods:**

A cross sectional study was conducted on a sample of 240 newborns at delivery ward. The collected data were cleaned, coded and entered into Epi -data version 4.2 and exported to Stata version 14. Binary logistic regression model was considered and statistical significance was declared at P< 0.05 using adjusted odds ratio.

**Results:**

The prevalence of asphyxia neonatorum was 6.7 % based on the fifth minute APGAR score. From multi-variable logistic regression analysis, antenatal obstetric complications (AOR = 2.63, 95% CI: 3.75, 14.29), fetal malpresentation (AOR = 3.17, 95% CI: 1.21, 15.20), premature rupture of fetal membranes (AOR = 6.56, 95% CI: 3.48, 18.12) and meconium stained amniotic fluid (AOR = 2.73, 95% CI: 1.76, 14.59) were significant predictors.

**Conclusion:**

The prevalence of fifth minute asphyxia neonatorum was relatively low. Fortunately, its predictors are modifiable. Thus, we can mitigate the problem even with our limited resources such as enhancing the existing efforts of antenatal and intra-partum care, which could help early detection and management of any obstetric and neonatal health abnormality.”

## Introduction

Asphyxia neonatorum or birth asphyxia is defined as “failure to initiate and sustain spontaneous breathing at birth 1, 2. It can also be defined as placental or pulmonary gas exchange impairment leading to hypoxemia and hypercarbia with a resultant effect of metabolic acidosis (evidenced by low umbilical cord pH< 7), low APGAR score less than 3 at 10^th^ minute and abnormal muscle tone 3–6. Besides, the American College of Obstetrics and Gynecology (ACOG) and American Academy of Pediatrics (AAP) defined birth asphyxia based on three (3) factors: metabolic or mixed acidemia determined from umbilical cord blood samples (pH<7), APGAR score of less than 3 for longer than five minute and multisystem organ dysfunction 5. The most valid diagnosis of birth asphyxia is made from the combination of three parameters. These are APGAR score less than 7, results of arterial blood gas analyses (PO^2^ and PCO^2^) and immediate newborns' umblical cord blood pH of < 7 6. However, in resource poor countries like Ethiopia where there is infeasibility of the latter two parameters, the parameter of Apgar score is used to diagnose birth asphyxia, evaluated in the fifth minute of life, with scores ranging from zero to ten. Thus, a diagnosis of birth asphyxia can be made when neonatal fifth minute Apgar score is less than 7. Apgar score values of four to seven indicate moderate birth asphyxia whereas severe asphyxia is between zero and three 7, 54.

Asphyxia neonatorum endangers neonatal survival. Severe degrees of birth asphyxia can cause severe neonatal multiorgan damage resulting in brain damage, lung dysfunction, cardiomyopathy, renal failure, hepatic failure and necrotizing enterocolitis 7–27. From these damages, brain damage is of the greatest concern becausvictim newborns are less likely to survive and even the survivors are more likely to have long-term neurological morbidities like cerebral palsy, permanent seizure disorder, intellectual incompetence and motor deficits, thereby leaving the survivors to be lifetime family burden. This in turn has raised the demand for costly technological care of asphyxiated newborns though the treatment outcome is often poor 3–5.

Asphyxia neonatorum can be caused by factors related to the antepartum, intra partum or post partum period^28^–^49^. However, quality of intrapartum care during labor and delivery has been recognized as the single most important predictor of the overall morbidity and mortality from asphyxia neonatorum 32, 34, 36–49. More specifically, factors like antenatal obstetric complications 17, 20,40, 42, 49,51–56, parity 19,42, multiple births 22, gestational age <37 or >41 weeks 22, 42, low birth weight 18, 40, 41, 42, premature rupture of membranes 20, 44, 45, 48, prolonged labor 18, 19, 41,42 and fetal distress 40,41, 48, 49 have already been identified to be among the risk factors of asphyxia neonatorum.

The Ethiopian neonatal mortality rate (NMR) is 29/1000 live births whilst Amhara region, a region of our study area, comprises the highest burden of the national NMR (47/1000 live births) 16. Literature shows asphyxia neonatorum is a universal public health problem with varied significance country wise 9, 15, 16. For example, 31.6% of the Ethiopian neonatal mortality is attributed to asphyxia neonatorum 24.

A significant number of neonatal deaths (33.3%) at Neonatal Intensive Care Unit (NICU) of Debre Tabor General Hospital (DTGH) are attributable to birth asphyxia. Most of these neonates are referred from maternity ward of the hospital. However, there is no recent evidence on the proportion and specific determinants of birth asphyxia at DTGH 50, 53. Moreover, the role of some important factors like birth spacing and birth weight for gestational age weren't addressed in the prior studies. Therefore, this study was aimed at determining the prevalence and specific predictors of birth asphyxia among newborns delivered at maternity ward of Debre Tabor General Hospital, Debre Tabor Town, South Gonder Zone, North Central Ethiopia, which is helpful to make interventions appropriate to the context of DTGH.

## Methods

### Study setting, period and design

A hospital-based quantitative cross sectional study was conducted from January first to April first 2019 at delivery ward of Debre Tabor General Hospital. The hospital is located in Debre Tabor town, 666 km far from Addis Ababa. It serves about 3,840 deliveries annually^50^.

### Study participants

Mothers who gave live birth after 28 weeks of gestational age were screened for eligibility. Newborns of unknown gestational age at birth were excluded. Furthermore, newborns with malformations incompatible to life such as hydrops and cyanotic congenital heart defects were excluded as these newborns have already been predisposed for asphyxia.

### Sample size determination and sampling technique

A total sample of 240 mother-newborn dyads was obtained using single population proportion formula; considering a confidence level of 95%, marginal error of 5%, a reasonable estimate for the proportion of asphyxia neonatorum from a previous study (p=0.829) 38 and adding a none response rate of 10%. The average monthly delivery rate of DTGH was 320 as per DTGH'S last quarter report of 2018. Then, to ensure representativeness of the calculated sample size (n=240), every fourth eligible mother newborn dyad was selected over 3 months at maternity ward of DTGH.

### Data collection procedure

A pretested interviewer based questionnaire was used to collect primary data on maternal socio-demographic and ante partum related factors. For twin births, every mother was asked only once about socio-demographic and antenatal factors of her twin babies because twin neonates share similar socio-demography and antenatal history. Furthermore, a pretested structured checklist was employed to abstract secondary data from maternal chart on intra-partum (induction and/augmentation, fetal distress, fetal presentation at birth, mode of delivery, fetal distress, time of membrane rupture, duration of labor, color of amniotic fluid) and neonatal related factors (sex, birth weight, gestational age at birth and birth weight to gestational ag). For twin births, every mother's chart was reviewed twice for the aforementioned intrapartum and neonatal related characteristics due to variation of these factors between twin neonates.

Fifth minute APGAR score was collected from direct observation by six trained BSc graduating class midwifery students both during day and night shifts in the delivery ward and operation room, which we got it feasible. Apgar score was measured using five components (Skin color / oxygenation; totally pinkish body (2 points), body pink but bluish extremities (1 point), totally blue or pale body (0 point)); (Pulse or heart rate ; ≥100 beats/minute (2 points), 100 (1 point), absent (0 point)); (Grimace or reflex irritability to stimuli; Crying or coughing or sneezing (2 points), grimacing (1 point), no response at all (0 point));(Activity or muscle tone and movement; well flexed or active motion (2 points), some flexion of extremities (1 point), flaccid or limp (0 point)) and (Respiration or breathing effort or lung maturity; (Good cry or regular breathing (2 points), weak or irregular and < 30 breath/minute (1 point), absent or apnea (0 point). Then, the score of each component was summed up by the data collectors and the information was documented on an Apgar score card (additional file 1).

The diagnosis of birth asphyxia was made based on fifth minute APGAR score.. The diagnoses of birth asphyxia were further confirmed through medical interns' consultation of obstetricians and pediatricians to deal with its severity and management.

The questionnaire and checklist were adapted from Ethiopian and other African studies 17–19, 20, 22, 40–42. The questionnaire was first prepared in English and translated to Amharic (local language to conduct interview) and retranslated to English to check for consistency. Before the actual data collection, pretest was done using 12 eligible mother- newborn dyads (5% of sample size) at the same hospital a week before data collection. One day training was first provided for data collectors and supervisors on the process of data collection.

### Operational definitions

**Birth asphyxia:** A newborn was considered to have birth asphyxia when its fifth minute APGAR score was <7 7, 54.

### Prolonged labor

**Prolonged labor (second stage):** For nulliparous mothers, if labor exceeds 3 hours with provision of regional anesthesia, or 2 hours without regional anesthesia. For multiparous mothers, if labor exceeds 2 hours with regional anesthesia or 1 hour in the absence of regional anesthesia. We have assessed this in numbers. 7. Prolonged labor was collected from the maternal chart. Premature rupture of membrane was diagnosed when fetal chorio-amnionic membranes rupture at any time before the onset of true labor 7.

### Statistical analysis

The collected data were coded, cleaned, edited and double entered into epidata version 4.2 after which it was exported to STATA version 14. Frequencies, proportion, summary statistics and cross tabulation were used to describe the study population. Using binary logistic regression model, bivariable analysis was first carried out to identify candidate variables (P<0.25) for multivariable analysis. Then, multivariable logistic regression analysis was performed to investigate statistically significant predictors of asphyxia neonatorum by adjusting for possible confounders. Finally, variables (P<0.05) were reported as statistically significant using AOR with 95% CI.

## Results

### Socio -demographic factors

In this study, the overall 240 respondents were involved thereby making a response rate of 100%. More than half of the respondents, 145 (60.4%) were rural residents. Moreover, 221(92.9%) of the respondents were married and concerning their educational status, about one third of them (32.4%) were unable to read and write. The mean maternal age was 28.70 (SD=± 5.86) years of whom about three quarters, 185(77%) were in the age group of (>34 years). Nearly half of the mothers, 125 (52.5%) have less than two years of birth spacing for the index newborn. Besides, about one quarter, 53 (22.1%) of the mothers had history of adverse pregnancy outcome of which prematurity 23(43.4%) accounted for the highest proportion (Table 1).

**Table 1:** Socio-demographic factors of mothers who gave live birth at Debre Tabor General Hospital, 2019 (n=240)

Factor	Response	n	%
Residence	Rural	145	60.4%
	Urban	95	39.6%
Age	15–19	15	6.3%
	20–34	40	16.7%
	>34	185	77.0%
Marital status	Married	221	92.1%
	Widowed	14	5.8%
	Separated	5	2.1%
Religion	Orthodox	221	92.1%
	Muslim	19	7.9%
Occupation	House wife	141	58.8%
	Governmental Employee	41	17.0%
	Merchant	16	6.7%
	Daily Labor	42	17.5%
Educational Status	Unable to read and write	77	32.1%
	No formal education but can read and write	78	32.5%
	Primary education (1–8)	42	17.5%
	Secondary education (9–12)	19	7.9%
	College or University	24	10%
Gravidity	<3	72	30
	≥3	168	70
Parity	Primiparous	61	25.4
	Multiparous	179	74.6
Birth spacing (Years)	< 2	125	52.1
	≥2	115	47.9
History of adverse pregnancy outcome	Yes	53	22.1
	No	187	77.9
Type of adverse pregnancy outcome ?(**n=53**)	Abortion	7	13.2
	Intrauterine fetal death	6	11.3
	Still birth	9	17.0
	Preterm	23	43.4
	Neonatal death	8	15.1

### Ante partum related factors

Nearly all the mothers, 233(95.8%) had attended antenatal care at public hospitals (59.7%) and public health center (40.3%). However, it was only about half of the mothers (52.6%) who had four and above antenatal care visits. About one tenth of the mothers (11.25%) ever used substance during their gestation of which ever alcohol users accounted for the most majority (70.4%). In the antenatal period, 87(36.3%) mothers were found to have obstetric complications. From these complications, preeclampsia/eclampsia accounted for the highest percentage (37.9%) followed by antepartum hemorrhage (23.0%), anemia (21.8%), infections (11.5%) and gestational diabetes (5.7%) (Table 2).

**Table 2:** Factors related to the ante partum period among mothers who gave live birth at Debre Tabor General Hospital, 2019 (n=240)

Factor	Response	N	%
ANC	Yes	233	95.8
	No	7	4.2
Number of ANC visits **(n=233)**	<4	112	48.1
	≥4	121	51.9
Obstetric complication during pregnancy	Yes	87	36.3
	No	153	63.8
Type of complication (**n=87**)	Preeclampsia/eclampsia	33	37.9
	Antepartum hemorrhage	20	23.0
	Anemia	19	21.8
	Infections	10	11.5
	Gestational diabetes	5	5.7
Ever used substance during pregnancy	Yes	27	11.25
	No	213	88.75
Type of substance ever used during pregnancy (**n=27**)	Alcohol	19	70.4
	Khat	6	22.2
	Cigarette	2	7.4

### Intra partum related factors

Of the total respondent mothers, 144 (60%) had spontaneous labor onset. Majority of the mothers 188(78.3%) had intrapartum rupture of fetal membranes whereas premature rupture of membrane was reported among 52 (21.7%) mothers. Following membrane rupture, meconium stained amniotic fluid was observed among 97 (40.4%) mothers and similar report was obtained to the presence of fetal distress 98 (40.8%). At labor, about one third of the fetuses 73(30.4%) were malpresented and nearly equal number of fetuses were delivered by cesarean section 71 (29.6%) (Table 3).

**Table 3:** Factors related to the intrapartum period among mothers who gave live birth at Debre Tabor General Hospital, 2019 (n=240)

Factor	Response	N	%
Fetal presentation	Vertex	167	69.6
	Malpresentation	73	30.4
Mal-presentation type (n=73)	Breech	52	71.2
	Face	21	28.8
Labor type	Spontaneous onset	144	60
	Induced	34	14.2
	Augmented	62	25.8
Labor duration	Normal	177	73.8
	Prolonged	44	18.3
	Precipitated	19	7.9
Time of membrane rupture	PROM	52	21.7
	Intrapartum	188	78.3
Duration of PROM until delivery occurs ***n=52***	Normal	39	75
	Prolonged	13	25
Color of amniotic fluid	Meconium stained	97	40.4
	Clear	143	59.6
Fetal distress	Yes	98	40.8
	No	142	59.2
Mode of delivery	SVD	139	57.9
	C/S	71	29.6
	Instrumental	30	12.5
Birth attendant	Midwife	142	59.2
	Emergency surgeon	48	20
	Obstetrician	23	9.6
	Medical intern	17	7.1

### Newborn related characteristics

More than half of the newborns, 132 (55%) were males. There were 34 twin newborns. The mean gestational age at birth was 38.3 (±2.3) weeks and majority of the newborns (64.2%) were term. Moreover, the mean birth weight was 2674.3 (±1484.2) grams and about one third of the newborns 73(30.4%) had low birth weight. When birth weight was considered against the corresponding gestational age at birth, about one fifth of the total newborns 53(22.1%) had birth weight inappropriate for their gestational age at birth. Of those 53(22.1%) newborns with inappropriate birth weight for gestational age, 24 (10%) were small for their gestational age whereas the remaining 29 newborns (12.1%) had large birth weight for their corresponding gestational age (Table 4).

**Table 4:** Newborn related characteristics at Debre Tabor General Hospital, 2019 (n=240)

Factor	Response	n	%
Sex	Male	132	55
	Female	108	45
Birth outcome	Singleton	206	85.8
	Twin	34	14.2
Birth weight	<2500	73	30.4
	≥2500	167	69.6
Gestational age at birth	Preterm	71	29.6
	Term	154	64.2
	Post term	15	6.3
Birth weight for Gestational age at birth	Appropriate for gestational age	187	77.9
	Small for gestational age	24	10.0
	Large for gestational age	29	12.1

### Proportion Prevalence of asphyxia neonatorum

**Figure 1:**
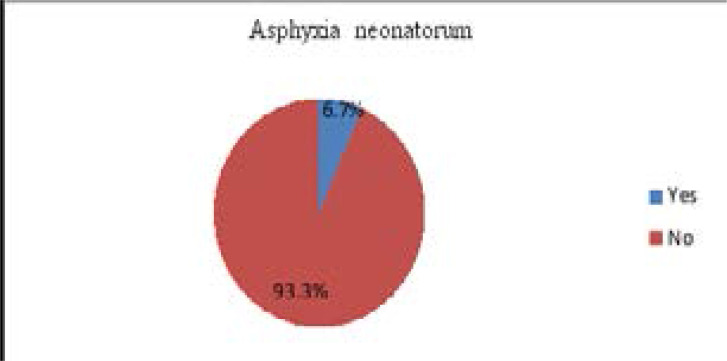
Prevalence of asphyxia neonatorum among live births at DTGH (n=240), 2019.

The prevalence of asphyxia neonatorum was found to be 16(6.7 %) (Fig 1). All cases of the birth asphyxia were of moderate severity.

Besides, first minute APGAR score showed that 99 (41.3%) neonates had less than 7 APGAR score. Most of these neonates, 96 (40.0 %) had

### Determinants of asphyxia neonatorum

The bivariable logistic regression analysis showed that antenatal obstetric complications, fetal malpresentation, premature rupture of fetal membranes, meconium stained amniotic fluid, fetal distress, mode of delivery and birth spacing were crudely associated with asphyxia neonatorum. However, after statistical adjustments for the likely effects of confounding variables, only four of the aforementioned factors namely antenatal obstetric complications, fetal malpresentation, premature rupture of fetal membranes and meconium stained amniotic fluid, were significant predictors of asphyxia. In this study, the width interval for three of the adjusted point estimates (premature rupture of membrane, fetal malpresentation and meconium stained amniotic fluid) is somewhat wider, thereby indicating a relatively less precise measurement of these factors. However, the width interval for the CIs of these factors is less than 17, which is in the recommended gap as shown in table 5 given below.

**Table 5:** Precision of the point estimates on the significant determinants of asphyxia neonatorum among live births at Debre Tabor General Hospital, North Central Ethiopia, 2019 n=240

Factor	Adjusted effect size estimate (AOR)	Lower bound	Upper bound	Width of CIs (i.e. Upper bound minus lower bound)
Premature rupture of membrane	6.56	3.48	18.12	14.64
Fetal mal presentation presentation	3.17	1.21	15.20	13.99
Meconium stainedamniotic fluid	2.73	1.76	14.59	12.83
Antenatal obstetric complications	2.63	3.75	14.29	10.54

The amendment to the original manuscript is included in the revised version manuscript as shown by the tracked insertions under the determinants of asphyxia neonatorum and limitation of the study on pages 9, 12 and 13.

The likelihood of developing birth asphyxia among neonates born to mothers with antenatal obstetric complications was 2.63 times (AOR = 2.63, 95% CI: 3.75, 14.29) more when compared to their counter-parts. Neonates born with fetal malpresentation had 3.17 times (AOR = 3.17, 95% CI: 1.21, 15.20) more likelihood of being asphyxiated at birth as compared to those of vertex presentations. Neonates born to mothers with premature rupture of fetal membranes were 6.56 times (AOR = 6.56, 95% CI: 3.48, 18.12) more prone to be asphyxiated at birth when compared to those with intrapartum rupture of membranes. Similarly, neonates born to mothers having meconium stained amniotic fluid were 2.73 times (AOR = 2.73, 95% CI: 1.76, 14.59) as likely to have birth asphyxia as compared to those born without being meconium stained.

## Discussion

Hypoxic-ischemic damage of neonatal vital organs mainly during the intrapartum period is the leading cause of mortality from asphyxia neonatorum. Thus, the quality of obstetric care at birth is crucial to reduce the overall newborn mortality and its long-term consequences 1, 2, 4, 6. In this study, it was tried to identify the predictors of asphyxia neonatorum among live births at Debre Tabor General Hospital. Antenatal obstetric complications, fetal malpresentation, premature rupture of fetal membranes, prolonged labor, meconium stained amniotic fluid, fetal distress and CS delivery were found to be significant predictors of asphyxia neonatorum.

“From this study, the prevalence of birth asphyxia was 6.7%, which was lower than a study in Iran (58.8%) 21. This may be due to differences in sample size and study setting. Besides, most importantly, difference in case definition might have played contribution for the variation; for example, our study was based on only fifth minute APGAR score less than 7 whereas that of the Iranian study used to have a flexible diagnostic criteria of birth asphyxia including: umbilical cord pH<7 or 5 min Apgar score <6 or 20 minute Apgar score less than 7 or multi organs failure in the first 72 hours or convulsion in the first 24 hours of life. The prevalence of birth asphyxia in our study was also lower than the study in Nigeria (21.1%)^19^ which may be attributed to the use of non APGAR score clinical parameters by the Nigerian study that included even out born neonates. Moreover, the prevalence of birth asphyxia at our study setting was lower than other prior Ethiopian studies at Dilla (32.8%) 17 and Jimma (47.5%) 51 that could be attributed to the relatively smaller sample size in our study and study setting difference (ours is at general hospital, while others were held at referral hospitals where more complicated and referral deliveries present with greater proportion of birth asphyxia than general hospitals).”

In the analysis of associated factors, the likelihood of developing birth asphyxia among neonates born to mothers with antenatal obstetric complications was 2.6 times higher as compared to their counter-parts. Different prior Ethiopian studies 17, 18, 40, 41, 42 revealed similar findings. Besides, the finding was consistent with other studies conducted in both developed and developing countries 19, 20, 43, 44, 46, 49 where the odds of birth asphyxia among neonates born to mothers with obstetric complications were about three to eleven times higher than those born to mothers who had no antenatal obstetric complications. This could be due to the fact that antenatal obstetric complications are often accompanied with feto placental hypoperfusion and subsequent ischemia that in turn cause intrauterine fetal hypoxia 3, 6, 52, 54

Neonates born with fetal malpresentation were 3.2 times more likely of being asphyxiated as compared to those with vertex presentations. This finding was congruent with other Ethiopian studies 18, 40 where the odds of asphyxia neonatorum among the malpresented fetuses were 6.98 times and 4.46 times higher as compared to the vertex presented fetuses respectively. A Cameroonian study 20 also showed similar finding. This similarity could be due to the fact that malpresentation is often associated with fetal life threatening complications like umbilical cord accidents (cord prolapse, cord compression) and prolonged labor 6.

Neonates born to mothers with premature rupture of fetal membranes were 6.6 times more prone to be asphyxiated at birth as compared to those with intrapartum rupture. A consistent finding was obtained from studies at Cameroon 20, Uganda 45 and Al-Diwaniya teaching hospital 48. The consistence can be justified by the fact that when fetal membranes rupture prematurely, spontaneous gush of amniotic fluid along with umbilical cord prolapse happens. Moreover, premature rupture of membranes, if prolonged, often facilitates feto-maternal systemic infections^3^, ^6^, ^7^, which is usually ensued by subsequent asphyxia neonatorum.

The risk of birth asphyxia among neonates with history of meconium stained amniotic fluid was 2.7 times higher than those born to mothers with clear amniotic fluid. This finding was consistent with other studies 17, 18, 40–42,45, 46, 47. The likely justification may be due to the fact that meconium stained amniotic fluid results in intrapartum inhalation of meconium which leads to mechanical obstruction of airways, surfactant inactivation, chemical inflammation and apoptosis of the pulmonary tissues thereby facilitating pulmonary air leak and hypoxia 6, 55.

From bivariable analysis, it has been shown that neonates born to mothers with birth spacing of < 2 years were 1.89 times more likely to be asphyxiated as compared to those with ≥2 years of birth spacing. This may be due to the fact that narrow birth spacing (< 2 years) is a universal public health problem having association with adverse maternal, fetal and neonatal outcomes such as low birth weight 56 ,57, preterm delivery, small for gestational age 58, precipitous labor 59, gestational diabetes 60, 61, anemia 61, 62, uterine rupture, premature rupture of membrane, preeclampsia and chronic hypertension 60, 63, 64. Significance of some of these outcomes is also supported by this study.

## Limitation of the study

The study lacks support of qualitative data. Moreover, the results may not be representative of the entire newborns in Ethiopia due to a small sample size consideration in this study. The study also shares drawbacks of a cross-sectional design.

Despite the aforementioned findings, the results are limited to the diagnosis of birth asphyxia based on solely fifth minute APGAR score. However, to minimize its effect on the validity of our estimate about the prevalence of birth asphyxia, the data collectors underwent efforts of consulting senior professionals (midwives and Integrated Emergency surgery obstetricians) about the neonatal condition to confirm the diagnosis of asphyxia. The study also shares drawbacks of a cross-sectional design. Furthermore, the study lacks support of qualitative data.

## Conclusion

Compared to prior studies at other settings, the prevalence of asphyxia neonatorum in our study area was relatively low. Presence of antenatal obstetric complications, fetal malpresentation, premature rupture of fetal membranes and meconium stained amniotic fluid were significantly associated with increased odds of asphyxia neonatorum. Therefore, early antenatal screening and follow up of every mother should be given due strict emphasis for timely detection and management of any obstetric complication. Besides, mothers with fetal malpresentation should be given thorough partographic follow up of their labor to early detect any fetal abnormality for emergency actions. Mothers with PROM should be provided with prophylactic antibiotics to prevent feto-neonatal systemic infections. Besides, digital vaginal examinations of PROM mothers should be kept as minimal as possible to reduce the likelihood of chorioamnionitis. Appreciating meconium stained amniotic fluid during labor and/delivery should be regarded as a signal of birth asphyxia; hence, delivery care providers have to be ready to give immediate resuscitation for such neonates.

## Figures and Tables

**Table 6:** Bivariable and multivariable logistic regression analysis of factors associated with asphyxia neonatorum among live births at Debre Tabor General Hospital, North Central Ethiopia, 2019 (n=240)

Factor	Birth asphyxia		COR(95%CI)	AOR(95%CI)
	Asphyxiated (n=16)	Not asphyxiated (n=224)		
**Antenatal** **obstetric complications**				
Yes	11 (68.8%)	76(33.9%)	4.88 (2.41, 9.32)	2.63 (3.75, 14.29)*
No	5(31.2%)	148 (66.1%)	1.0	1.0
**Fetal presentation**				
Vertex	12 (75.0%)	155(69.2%)	1.0	1.0
Malpresentation	4 (25.0%)	69 (30.8%)	1.34 (1.13, 10.33)	3.17 (1.21, 15.20)*
**Time of** **membrane rupture**				
PROM	7(43.8%)	40 (17.9%)	3.58(2.44, 8.27)	6.56(3.48, 18.12)*
Intra-partum	9 (56.2%)	184 (82.1%)	1.0	1.0
**Color of amniotic fluid**				
Meconium stained	9 (56.2%)	88 (39.3%)	1.99 (4.70, 15.27)	2.73 (1.76, 14.59)*
Clear	7 (43.8%)	136 (60.7%)	1.0	1.0
**Fetal distress**				
Yes	8 (50.0%)	90 (%)	1.49 (1.37, 19.28)	0.51 (0.89, 2.12)
No	8 (50.0%)	134 (%)	1.0	1.0
**Mode of delivery**				
SVD	7(43.8%)	132 (58.9%)	1.0	1.0
Instrumental	5 (31.2%)	25 (11.2%)	0.27 (0.14, 0.43)	0.51 (0.87,13.40)
CS	4 (25.0%)	67 (29.9%)	0.89 (0.42, 0.97)	0.67 (0.29, 8.33)
**Birth spacing**				
≥2 years	10(62.5%)	105(46.9%)	1.0	1.0
<2 years	6(37.5%)	119(53.1%)	1.89 (1.30, 12.88)	3.58(0.79, 10.24)

* Statistically significant at P<0.05, COR (Crude Odds Ratio), AOR (Adjusted Odds Ratio)

## Abbreviations

WHO: World Health Organization; ANC: Ante Natal Care; APGAR: Appearance, Pulse Rate, Grimace, Activity, Respiration Rate; CS: Cesarean Section; DTGH: Debre Tabor General Hospital; EDHS: Ethiopian Demographic Health Survey; FMOH: Federal Ministry of Health; GA: Gestational Age; NICU: Neonatal Intensive Care Unit; NMR: Neonatal Mortality Rate; PROM: Premature Rupture of Membrane; SVD: Spontaneous Vaginal Delivery

## Declarations

### Availability of data and materials

Data will be available upon request from the corresponding author.
